# Dormant Crohn's Disease Reactivated by Clostridioides difficile Infection

**DOI:** 10.7759/cureus.37062

**Published:** 2023-04-03

**Authors:** Oscar L Hernandez, Zoilo K Suarez, Talwinder Nagi, Charles Vallejo, Allison Ferris

**Affiliations:** 1 Internal Medicine, Florida Atlantic University Charles E. Schmidt College of Medicine, Boca Raton, USA

**Keywords:** clostridioides difficile, crohn’s disease, colonoscopy, intestinal microbiota, humoral immunity, acute infectious diarrhea, crohn’s disease (cd), inflammatory bowel disease, clostridioides difficile infection

## Abstract

Crohn’s disease (CD) is a type of inflammatory bowel disease (IBD) characterized by chronic transmural inflammation of any portion of the gastrointestinal tract. The etiology of CD remains unknown although genetic, immunological, and acquired factors have been recognized as contributing to its development. Alterations of intestinal microbiota, including *Clostridioides difficile* (*C. difficile*), are theorized to alter humoral immunity and contribute toward CD flare pathogenesis. As such, cases of IBD remission can be undone by alterations in the gut microbiota and subsequently confound the diagnosis of inflammatory or infectious etiologies of diarrhea. We present a case of a 73-year-old female with dormant CD for 25 years who experienced an atypical course of diarrhea found to have a CD flare in the setting of acute *C. difficile* colitis.

## Introduction

Crohn’s disease (CD) is a type of inflammatory bowel disease (IBD) characterized by chronic transmural inflammation of the gastrointestinal tract, which may lead to the formation of fistulas, obstruction, or perforation [1]. As described by Xu et al., the recognition of CD is increasing as reflected by a rising prevalence in the United States [2]. While its precise pathophysiology remains unknown, there are numerous genetic, immunological, and acquired factors that are hypothesized to play a role in the development of CD [3]. Alterations of the microbiota of the intestine [4], including the presence of *Clostridioides difficile* (*C. difficile*), have been thought to be part of the pathogenesis of CD [5]. Clayton et al. have demonstrated a significant association between the asymptomatic carriage of *C. difficile* and IBD when compared to healthy volunteers [6]. Furthermore, *C. difficile* colitis has been associated with increased morbidity, length of stay, and incidence of flares in patients suffering from IBD [7-9]. Distinguishing an acute flare of IBD in the setting of an active *C. difficile* colitis remains especially challenging [10,11]. We present a case of a 73-year-old female with a history of dormant CD with subsequent flare due to an acute *C. difficile* infection requiring endoscopic evaluation for diagnostic differentiation.

## Case presentation

A 73-year-old female with a history of CD in remission for 25 years without maintenance therapy presented to an academic health center for evaluation of progressively worsening nausea, vomiting, and diarrhea onset three days prior. She reported her symptoms were gradual onset and associated with severe non-bloody malodorous watery diarrhea. The patient denied recent sick contacts, recent travel, or restaurant exposure. Additionally, she denied any associated fevers, rashes, or recent antibiotics exposure in the two months prior. Due to her aversion to oral intake from her symptoms and lethargy, she was admitted for further evaluation and management.

On physical examination, the patient was afebrile with a temperature of 37.6°C, pulse rate of 75 beats per minute, blood pressure of 128/82 mmHg, and oxygen saturation of 96% on room air. Abdominal examination yielded normoactive bowel sounds, mild non-tympanic abdominal distention, and mild tenderness of the bilateral lower quadrants without guarding or rebound tenderness. Initial laboratory testing, as reported in Table 1, was notable for an elevated serum leukocyte count, erythrocyte sedimentation rate, and fecal calprotectin. *C. difficile* polymerase chain reaction testing was positive for toxigenic strains but enzyme-linked immunosorbent assay testing was negative for the presence of detectable toxins. Abdominal and pelvic computed tomography with intravenous contrast showed signs of feculent conversion of the distal small bowel without evidence of colitis.

**Table 1:** Significant laboratory values at presentation and throughout admission. *^a^* Polymerase chain reaction. *^b^* Enzyme-linked immunosorbent assay.

Variable	On admission	Day 2	Day 4	Day 5	Reference range
Serum leukocyte count	15.0 x 10^3^/μL	10.4 x 10^3^/μL	16.6 x 10^3^/μL	20.9 x 103/μL	4.5-11.0 x 10^3^/μL
Erythrocyte sedimentation rate	61 mm/hr	-	-	-	1-13 mm/hr
Fecal calprotectin	149 μg/mg	-	-	203 μg/mg	10-50 μg/mg
Fecal leukocyte count	Occasional	-	-	-	Absent
*C. difficile* gene PCR^a^	Positive	-	-	-	Negative
*C. difficile* toxin assay^b^	Negative for toxins A and B	-	-	-	Negative

Given that *C. difficile* testing could be confounded in the setting of underlying IBD, the top differential remained *C. difficile* infection despite a negative *C. difficile* toxin assay. She was started on oral vancomycin and the progression of her bowel movements and symptoms were closely monitored. By admission day three, her serum leukocyte count and abdominal pain had improved. She reported improvement in her diarrhea with a reduction in her bowel movement frequency. Additionally, her bowel movements began to become more formed with a less watery appearance.

**Figure 1:**
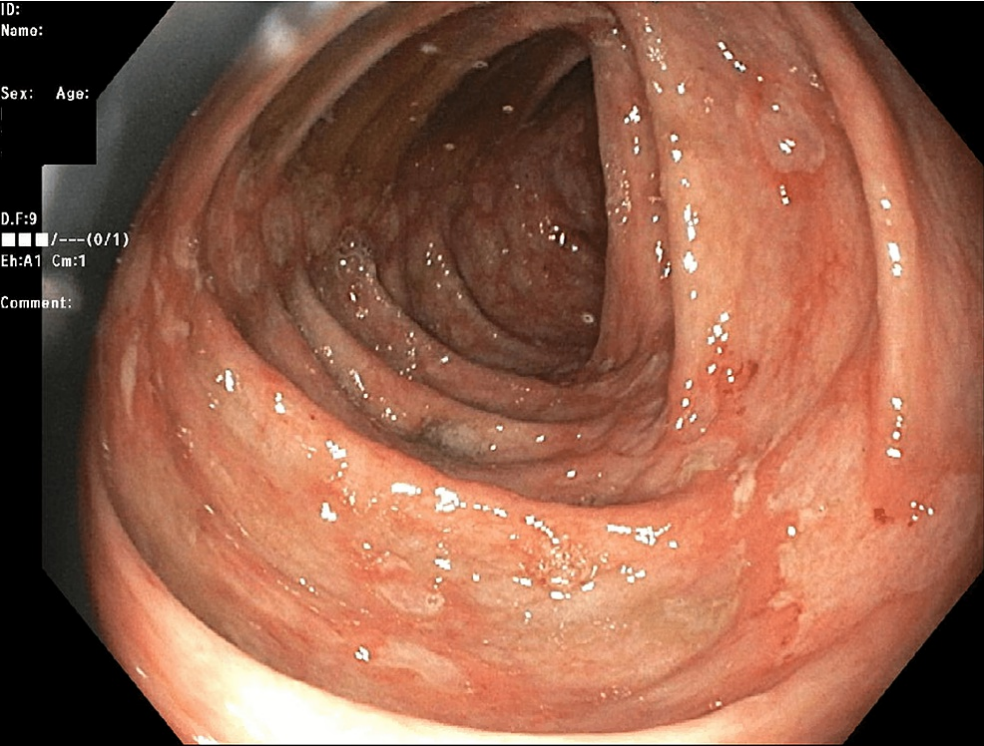
Colonoscopy showing numerous shallow ulcerations along the transverse colon without evidence of pseudomembranes.

**Figure 2:**
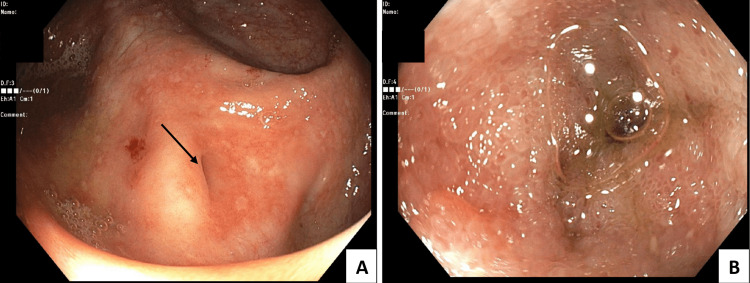
Colonoscopy showing lesions throughout the ascending colon (A) leading to the ileocecal valve (black arrow). View of the terminal ileum (B) following intubation of the ileocecal valve showing visible inflammation consistent with Crohn's disease.

Despite her initial improvement, she began to complain of worsening generalized abdominal pain associated with an increase in the frequency of her bowel movements on admission day four. Her serum leukocyte count acutely increased, as demonstrated in Table 1, while she lacked evidence of infectious sequelae besides her gastrointestinal symptoms. A repeat abdominal exam yielded more localized right lower quadrant pain in comparison to the bilateral lower abdominal pain on presentation. Repeat fecal leukocyte count was elevated as compared to her admission baseline. A gastroenterologist was consulted for further evaluation of the patient's atypical course of diarrhea due to suspicions of *C. difficile* colitis leading to a CD flare. To further guide treatment decisions, it was decided to conduct a colonoscopy for direct evaluation of the colon and differentiate these two etiologies. Diffuse, shallow ulcerations were noted throughout the colon (Figure 1) without evidence of pseudomembranous colitis. The terminal ileum was intubated showing evidence of diffuse ulcerations and inflammation (Figure 2).

Ultimately, these findings were consistent with a new Crohn’s flare. Intravenous methylprednisolone was initiated in addition to maintaining the patient’s oral vancomycin. The patient’s leukocytosis and abdominal pain both improved with complete resolution by admission day eight. She was discharged with oral prednisone and a 14-day course of oral vancomycin to continue maintaining her remission while following up with her gastroenterologist for further monitoring. Upon re-evaluation 24 days after discharge, her symptoms had resolved following the completion of both a 14-day course of oral prednisone and oral vancomycin. The patient elected against further pharmacotherapy for maintenance of remission. She was due to follow up with her gastroenterologist for a repeat colonoscopy five weeks after her discharge date.

## Discussion

*C. difficile* colitis has been associated with IBD flares in addition to increased morbidity, mortality, length of stay, and rates of colectomy among patients with IBD [7-9]. Although *C. difficile* colitis has been associated with IBD in this capacity, it can be difficult to discern between acute *C. difficile* colitis or a new onset IBD flare as clinical presentation can overlap. Additionally, previous studies have demonstrated the capacity for skewed *C. difficile* testing in patients with underlying CD [12]. Interpretation often requires consideration of clinical context as false negative testing for *C. difficile* can occur. Moreover, *C. difficile* is known to colonize patients with IBD and may produce IBD flares in the absence of detectable *C. difficile* toxin strains [12].

The association between CD and *C. difficile* colitis has been studied for decades [13]. *C. difficile* colitis is considered one of the most common complications and an important cause of flares in patients with IBD [5]. However, the exact mechanism by which *C. difficile* colitis induces CD flares is still yet to be elucidated. *C. difficile* has been hypothesized to damage the mucosa, induce cytokine release, and serve as a microbial-associated molecular pattern (MAMP) that activates toll-like receptor 4 (TLR-4). This triggers the nuclear factor kappa beta (NF-κB) and *HMGB1* pathways, resulting in the induction of both innate and adaptive immune responses [14,15]. Other cytokines, including interleukins 1β, 6, and 8, in addition to tumor necrosis factor-α and leukotriene B-4, have also been thought to play a role in this association [16]. The interleukin 4 gene-associated single nucleotide polymorphism rs2243250 has been suggested to provide a genetic component contributing toward acquiring the infection [17]. An abnormal immune response of immunoglobulin G against toxin B has been proposed to be responsible for the hyper-reactivity of *C. difficile* colitis in patients with IBD [18].

We postulate that these factors contributed toward inducing a CD flare in our patient. The patient's initial improvement of symptoms and leukocytosis with oral vancomycin therapy supports the diagnosis of *C. difficile* colitis as her initial condition instead of a flare of CD. Upon further review of our case, we suspect our patient's acute *C. difficile* colitis subsequently triggered an inflammatory response in the patient's gastrointestinal mucosal surfaces and thus induced a CD flare. Treatment for patients with a suspected CD flare in the setting of *C. difficile* colitis revolves primarily around treating the underlying bacterial infection with antibiotics while including anti-inflammatory measures such as systemic glucocorticoids to induce remission [16].

## Conclusions

In patients with dormant CD, *C. difficile* colitis may induce a flare with presenting symptoms of abdominal pain and diarrhea. The similarities between *C. difficile* colitis and active CD may confound practitioners and present as an atypical course of diarrhea. A high index of suspicion for a CD flare during a *C. difficile* infection is essential to enable expeditious evaluation and reduce the complications of *C. difficile* colitis associated with CD patients. An endoscopic evaluation may be utilized to differentiate these two etiologies or the presence of both simultaneously. Treatment for patients with *C. difficile* colitis and a CD flare includes the use of systemic corticosteroids and antibiotics with close monitoring of clinical progression.

## References

[1] McGregor CG, Tandon R, Simmons A (2023). Pathogenesis of fistulating Crohn's disease: a review. Cell Mol Gastroenterol Hepatol.

[2] Xu F, Carlson SA, Liu Y, Greenlund KJ (2021). Prevalence of inflammatory bowel disease among Medicare fee-for-service beneficiaries - United States, 2001-2018. MMWR Morb Mortal Wkly Rep.

[3] de Souza HS, Fiocchi C, Iliopoulos D (2017). The IBD interactome: an integrated view of aetiology, pathogenesis and therapy. Nat Rev Gastroenterol Hepatol.

[4] Nakase H, Uchino M, Shinzaki S (2021). Evidence-based clinical practice guidelines for inflammatory bowel disease 2020. J Gastroenterol.

[5] Sehgal K, Yadav D, Khanna S (2021). The interplay of Clostridioides difficile infection and inflammatory bowel disease. Therap Adv Gastroenterol.

[6] Clayton EM, Rea MC, Shanahan F, Quigley EM, Kiely B, Hill C, Ross RP (2009). The vexed relationship between Clostridium difficile and inflammatory bowel disease: an assessment of carriage in an outpatient setting among patients in remission. Am J Gastroenterol.

[7] Ananthakrishnan AN, McGinley EL, Saeian K, Binion DG (2011). Temporal trends in disease outcomes related to Clostridium difficile infection in patients with inflammatory bowel disease. Inflamm Bowel Dis.

[8] Ananthakrishnan AN, McGinley EL, Binion DG (2008). Excess hospitalisation burden associated with Clostridium difficile in patients with inflammatory bowel disease. Gut.

[9] Khanna S, Shin A, Kelly CP (2017). Management of Clostridium difficile infection in inflammatory bowel disease: expert review from the Clinical Practice Updates Committee of the AGA Institute. Clin Gastroenterol Hepatol.

[10] Boeriu A, Roman A, Fofiu C, Dobru D (2022). The current knowledge on Clostridioides difficile infection in patients with inflammatory bowel diseases. Pathogens.

[11] Del Vecchio LE, Fiorani M, Tohumcu E (2022). Risk factors, diagnosis, and management of Clostridioides difficile infection in patients with inflammatory bowel disease. Microorganisms.

[12] Dalal RS, Allegretti JR (2021). Diagnosis and management of Clostridioides difficile infection in patients with inflammatory bowel disease. Curr Opin Gastroenterol.

[13] Dorman SA, Liggoria E, Winn WC Jr, Beeken WL (1982). Isolation of Clostridium difficile from patients with inactive Crohn's disease. Gastroenterology.

[14] Petagna L, Antonelli A, Ganini C (2020). Pathophysiology of Crohn's disease inflammation and recurrence. Biol Direct.

[15] Yu F, Li X (2019). Impact of Clostridium difficile infection on immune function in patients with ulcerative colitis and the clinical nursing observation. Int J Clin Exp Med.

[16] Nitzan O, Elias M, Chazan B, Raz R, Saliba W (2013). Clostridium difficile and inflammatory bowel disease: role in pathogenesis and implications in treatment. World J Gastroenterol.

[17] Connelly TM, Koltun WA, Sangster W (2014). An interleukin-4 polymorphism is associated with susceptibility to Clostridium difficile infection in patients with inflammatory bowel disease: results of a retrospective cohort study. Surgery.

[18] Henriksson G, Bredberg J, Wullt M, Lyrenäs E, Hindorf U, Ohlsson B, Grip O (2019). Humoral response to Clostridium difficile in inflammatory bowel disease, including correlation with immunomodulatory treatment. JGH Open.

